# Supplementary material for: Filament formation by metabolic enzymes is a specific adaptation to an advanced state of cellular starvation
Source: eLife. 2014 Apr 25;3:e02409. doi: 10.7554/eLife.02409 (PMC4011332; doi:10.7554/eLife.02409)
Supplement: Supplementary file 1. — (A) Plasmids used in this study. (B) Antibodies used in this study. (C) Yeast strains used in this study. DOI: http://dx.doi.org/10.7554/eLife.02409.034 [file elife02409s001.doc]

# Supplemental file 1

**Filament formation by metabolic enzymes is a specific adaptation to an advanced state of cellular starvation**

**Ivana Petrovska, Elisabeth Nüske, Matthias C. Munder, Gayathrie Kulasegaran, Liliana Malinovska, Sonja Kroschwald, Doris Richter, Karim Fahmy, Kimberley Gibson, Jean-Marc Verbavatz, Simon Alberti**

Supplementary File 1A: Plasmids used in this study.

| **#** | **Accession number** | **Plasmid name** |
| --- | --- | --- |
| **1** | O-2446 | pAG415ADH1-GLN1 |
| **2** | O-2470 | pAG415GPD-GLN1-mCherry |
| **3** | O-2514 | pAG415GPD-GLN1(E186K)-mCherry |
| **4** | O-2517 | pAG415GPD-GLN1(P83R)-mCherry |
| **5** | O-2479 | pAG415GPD-GLN1(T49E)-mCherry |
| **6** | O-2529 | pAG415GPD-GLN1(Y81A)-mCherry |
| **7** | O-2520 | pAG415GPD-GLN1(R23E)-mCherry |
| **8** | O-2708 | pAG415GPD-GLN1(R23E,T49E)-mCherry |
| **9** | O-3048 | pAG415GPD-6xHIS-GLN1-V5 |
| **10** | O-3049 | pAG415GPD-6xHIS-GLN1(R23E)-V5 |
| **11** | O-3050 | pAG415GPD-6xHIS-GLN1(P83R)-V5 |
| **12** | O-2948 | pAG415GPD-6xHIS-GLN1-mCherry |
| **13** | O-2949 | pAG415GPD-6xHIS-GLN1(R23E)-mCherry |
| **14** | O-2950 | pAG415GPD-6xHIS-GLN1(P83R)-mCherry |
| **15** | O-2915 | pAG415ADH1-GLN1(T49E) |
| **16** | O-2918 | pAG415ADH1-GLN1(R23E) |
| **17** | O-2921 | pAG415ADH1-GLN1(R23E,T49E) |
| **18** | O-3051 | pAG415ADH1-GLN1-4Cys |
| **19** | O-3056 | pDEST24-GLN1 |
| **20** | O-3057 | pDEST24-GLN1(E186K) |
| **21** | O-3058 | pDEST24-GLN1(R23E) |
| **22** | O-2996 | pAG415GAL-GLN1-mCherry |
| **23** | O-3052 | pAG415GPD-GLN1-V5 |
| **24** | O-3053 | pAG415GPD-GLN1(R23E)-V5 |
| **25** | O-3054 | pAG415GPD-GLN1(P83R)-V5 |

Supplementary File 1B: Antibodies used in this study.

| **#** | **Antibody** | **Company name** | **Catalog number** |
| --- | --- | --- | --- |
| **1** | Anti-His6x tag | Dianova | Dia 900 |
| **2** | Anti-V5 tag | Invitrogen | R960-25 |
| **3** | Monoclonal anti-mCherry | In house | N/A |

Supplementary File 1C: Yeast strains used in this study.

| **#** | **Number** | **Background** | **Genotype** |
| --- | --- | --- | --- |
| 1 | 1928 | BY4741 | GLN1::GFP-HisMX |
| 2 | 2474 | W303 *ADE+* | GLN1::mCherry-KanMX |
| 3 | 2568 | W303 *ADE+* | gln1::SpHIS5, pAG416ADH1-GLN1 |
| 4 | 2716 | W303 *ADE+* | gln1::SpHIS5, pAG415GPD-GLN1-mCherry |
| 5 | 2972 | W303 *ADE+* | gln1::SpHIS5, pAG415GPD-GLN1(E186K)-mCherry |
| 6 | 2977 | W303 *ADE+* | gln1::SpHIS5, pAG415GPD-GLN1(P83R)-mCherry |
| 7 | 2975 | W303 *ADE+* | gln1::SpHIS5, pAG415GPD-GLN1(T49E)-mCherry |
| 8 | 2711 | W303 *ADE+* | gln1::SpHIS5, pAG415GPD-GLN1(Y81A)-mCherry |
| 9 | 2708 | W303 *ADE+* | gln1::SpHIS5, pAG415GPD-GLN1(R23E)-mCherry |
| 10 | 2973 | W303 *ADE+* | gln1::SpHIS5, pAG415GPD-GLN1(R23E,T49E)-mCherry |
| 11 | 3115 | W303 *ADE+* | gln1::SpHIS5, pAG415GPD-6xHIS-GLN1-mCherry |
| 12 | 3117 | W303 *ADE+* | gln1::SpHIS5, pAG415GPD-6xHIS-GLN1(R23E)-mCherry |
| 13 | 3121 | W303 *ADE+* | gln1::SpHIS5, pAG415GPD-6xHIS-GLN1(P83R)-mCherry |
| 14 | 3139 | W303 *ADE+* | URA8::sfGFP(V206R)-KanMX |
| 15 | 3028 | W303 *ADE+* | GLT1::sfGFP(V206R)-KanMX |
| 16 | 3141 | W303 *ADE+* | GCN3::sfGFP(V206R)-KanMX |
| 17 | 3010 | W303 *ADE+* | gln1::SpHIS5, pAG415ADH1-GLN1 |
| 18 | 3011 | W303 *ADE+* | gln1::SpHIS5, pAG415ADH1-GLN1(T49E) |
| 19 | 3247 | W303 *ADE+* | gln1::SpHIS5, pAG415ADH1-GLN1(R23E) |
| 20 | 3015 | W303 *ADE+* | gln1::SpHIS5, pAG415ADH1-GLN1(R23E,T49E) |
| 21 | 3019 | W303 *ADE+* | gln1::SpHIS5, pAG415ADH1-GLN1(P83R) |
| 22 | 3014 | W303 *ADE+* | gln1::SpHIS5, pAG415ADH1-GLN1(T81A) |
| 23 | 3245 | Prototrophic W303 | gln1::KanMX, ura3::HygB, pAG416ADH1-GLN1 |
| 24 | 3259 | Prototrophic W303 | gln1::KanMX, pAG41NAT-ADH1-GLN1 |
| 25 | 3260 | Prototrophic W303 | gln1::KanMX, pAG41NAT-ADH1-GLN1(R23E) |
| 26 | 2883 | Prototrophic W303 | Wildtype (Klosinska et al., 2011) |
| 27 | 3471 | Prototrophic W303 | GLN1::Y81A |
| 28 | 3465 | W303 *ADE+* | gln1::SpHIS5, pAG415GPD-6xHis-GLN1-V5 |
| 29 | 3466 | W303 *ADE+* | gln1::SpHIS5, pAG415GPD-6xHis-GLN1(R23E)-V5 |
| 30 | 3467 | W303 *ADE+* | gln1::SpHIS5, pAG415GPD-6xHis-GLN1(P83R)-V5 |
| 31 | 3468 | W303 *ADE+* | gln1::SpHIS5, pAG415GPD-GLN1-V5 |
| 32 | 3469 | W303 *ADE+* | gln1::SpHIS5, pAG415GPD-GLN1(R23E)-V5 |
| 33 | 3470 | W303 *ADE+* | gln1::SpHIS5, pAG415GPD-GLN1(P83R)-V5 |
